# E-cigarette or Vaping Product Use-Associated Lung Injury Complicated by Pulmonary Aspergillosis

**DOI:** 10.7759/cureus.20075

**Published:** 2021-12-01

**Authors:** Chloe Kupelian, Andrew Kim, Vini Vijayan

**Affiliations:** 1 Pediatrics, Valley Children’s Healthcare, Madera, USA

**Keywords:** pulmonary aspergillosis, ards, evali, vaping, e-cigarette

## Abstract

Since the initial reports regarding the nationwide outbreak of e-cigarette or vaping product use-associated lung injury (EVALI) in August 2019 by the Centers for Disease Control and Prevention, a clear link has been established between EVALI and tetrahydrocannabinol (THC)-containing product use. We report a case of invasive pulmonary aspergillosis (IPA) as a complication of EVALI in an immunocompetent adolescent that resulted in a fatal outcome. We encourage physicians that are considering the diagnosis of EVALI be cognizant of the increased use of THC and other potential contaminants in vaping cartridges. IPA can be a fatal disease and early aggressive treatment is necessary.

## Introduction

*Aspergillus spp*. is a ubiquitous filamentous mold found in soil and decaying vegetation. Inhalation of airborne conidia and hyphal fragments can cause life-threatening disease in immunocompromised patients with limited host defenses, such as those with solid organ transplants, hematologic malignancies, and immunodeficiencies. In immunocompetent hosts, invasive pulmonary aspergillosis (IPA) is rare but has been described in patients with risk factors such as cirrhosis, burns, and influenza infection [1]. Studies have been published regarding the development of IPA among immunocompromised individuals that reported medicinal marijuana use [2].

We report a case of invasive pulmonary aspergillosis as a complication of e-cigarette or vaping product use-associated lung injury (EVALI) in an immunocompetent adolescent that resulted in a fatal outcome.

This case report was presented as an oral presentation at the 2021 Virtual Western Medical Research Conference on January 29-30, 2021.

## Case presentation

A 16-year-old male presented to the emergency department (ED) with a one-week history of fever, cough, dyspnea, and chest pain. Upon arrival in the ED, his temperature was 37.8°C, pulse 117 bpm, blood pressure 116/77, respiratory rate 44 breaths per minute, and pulse oximetry 70% on room air. On examination, the patient was in severe respiratory distress, and auscultation of his lungs revealed decreased breath sounds bilaterally. He was tachycardic with a capillary refill of 4-5 seconds. The remainder of his examination was within normal limits.

Laboratory evaluation was significant for a white blood cell count (WBC) of 12,400/uL, (75% neutrophils, 2% eosinophils), and hemoglobin 14.2 g/dL. The viral respiratory panel was negative. Chest radiograph showed bilateral hazy ground-glass opacities. He was started empirically on ceftriaxone, vancomycin, and azithromycin for suspected community-acquired pneumonia. Blood cultures and sputum cultures were negative. The patient was placed on Bilevel Positive Airway Pressure (BiPAP) for respiratory support.

On hospital day 6, the patient acutely decompensated requiring emergent intubation and mechanical ventilation. Further history revealed that he occasionally used electronic cigarettes (e-cigarettes). There was no history of intravenous drug use or sexual activity, sick contacts, animal exposure or travel. His parents were unable to provide further details regarding the frequency or source of e-cigarette use.

**Figure 1:**
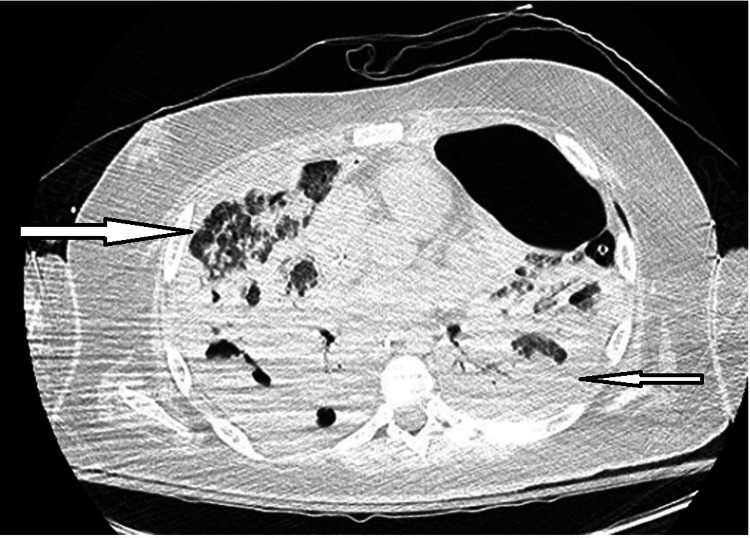
CT scan of the chest demonstrating diffuse ground-glass opacities in the upper lung fields and near-complete opacification of the lower lobes bilaterally with multifocal areas of cystic necrosis and cavitary lesions.

Computed tomography of the chest showed bilateral pleural effusions, areas of diffuse ground-glass opacities in the upper lung fields, cystic necrosis and bibasilar cavitations (Figure 1). 

*Legionella *urine antigen, serum* Histoplasma* antigen, serum* Blastomyces* antigen were negative. *Cryptococcus neoformans*, *Coccidioidomycosis*, *Blastomyces*, and *Histoplasma*, and human immunodeficiency virus were negative. His QuantiFERON-TB Gold was negative. Rheumatologic workup including antinuclear antibody, anti-neutrophil cytoplasmic antibody, proteinase 3 antibodies, antiphospholipid antibodies, anti-glomerular basement membrane antibodies were also negative. His urine drug screen was positive for tetrahydrocannabinol (THC). He underwent a bronchoscopy with bronchoalveolar lavage (BAL) revealing blood-tinged secretions, 9200 RBC/mcL, 85% neutrophils, and alveolar macrophages positive for lipid in Oil-O-Red stain concerning for e-cigarette or vaping product use-associated lung injury (EVALI).

The patient continued to deteriorate with worsening oxygenation and hypercarbia prompting transition to high-frequency oscillatory ventilation. Despite optimizing ventilator settings, he continued to have persistent hypoxemia, requiring initiation of venous-venous extracorporeal membrane oxygenation on hospital day 7. Laboratory values at that time included WBC count of 42,800 cells/uL with 90% neutrophils, and C-reactive protein of 30.9 mg/dL. His antimicrobial regimen was broadened to meropenem, vancomycin, clindamycin, levofloxacin. Methylprednisolone was also initiated for likely EVALI. As the child resided in San Joaquin Valley, liposomal amphotericin B was added for possible coccidioidomycosis.

On hospital day 11, BAL cultures confirmed growth of *Aspergillus niger* indicative of invasive pulmonary aspergillosis (IPA) and therefore, voriconazole and caspofungin were added to his antimicrobial regimen. On hospital day 40, the patient acutely developed pulmonary hemorrhage and despite inotropic support and transfusion of blood products, he succumbed to his illness.

## Discussion

Since the initial reports regarding the nationwide outbreak of EVALI in August 2019 by the Centers for Disease Control and Prevention (CDC), a clear link has been established between EVALI and tetrahydrocannabinol (THC)-containing product use [2]. Nationwide, 82% of patients hospitalized with EVALI endorsed THC-containing product use. As of February 2020, 2,807 cases of hospitalized EVALI or death have been reported to the CDC. Of these cases, 15% occurred in patients under 18 years of age [2]. Of these patients, 78% reported obtaining their supplies from informal sources including friends, online vendors and unregulated dispensaries. The practice of “dabbing” has become increasingly popular amongst youth and encompasses heating substances with a high concentration of THC in order to generate an extremely concentrated aerosol [2]. This practice significantly increases the risk of developing EVALI and secondary infections [3]. Although we were unable to determine the source of our patient’s supply and whether or not he practiced dabbing, we suspect he acquired products through an unregulated source.

E-cigarettes often contain harmful additives such as propylene glycol and vegetable glycerin, which when inhaled, create carbonyl compounds, leading to an inflammatory cascade and oxidative stress in the lungs. The subsequent inflammation has been shown to cause airway epithelial injury, disturbances in mucociliary clearance, increased risk for infection, and impairment of gas exchange [3, 4]. Unregulated THC-containing products are particularly harmful given the reports of contamination of the vaping liquids and cartridges with bacteria and mold. Studies have found that the possible constituents responsible for causing EVALI include vitamin E acetate, medium-chain triglyceride oil, and butane hash oil [4, 5, 6]. We did not attempt to test for these contaminants during our patient’s BAL; however, he did meet CDC criteria for EVALI based on his clinical presentation and BAL fluid composition.

Our patient had a history of THC use and vaping and although we did not have access to the cartridge that he used, based on his history, imaging and BAL cultures, he most likely developed IPA due to vaping THC compounds. While our patient had no underlying immunocompromising conditions, his vaping of THC may have increased his susceptibility to developing IPA and consequently pulmonary hemorrhage from this angioinvasive organism.

## Conclusions

To our knowledge, this is the first report to suggest IPA as a complication of EVALI in an immunocompetent adolescent. E-cigarette or vaping of nicotine and marijuana-based products is associated with a number of complications including inhalation of toxic contaminants, particularly when using counterfeit products, chemical pneumonitis and life-threatening hypoxemia. Critically ill patients with vaping-induced lung injury are at risk for serious secondary bacterial and fungal infections and death. With the rising popularity of vaping among middle school and high school students, it is critical that physicians be aware of this complication of vaping.

Our case emphasizes another potential risk associated with vaping. We encourage physicians that are considering the diagnosis of EVALI to be cognizant of the increased use of THC and other potential contaminants in vaping cartridges. IPA can be a fatal disease and early aggressive treatment is necessary. In critically ill patients who are not responding to antimicrobial therapy, consider testing and broadening coverage for fungal etiologies.
